# Supplementary material for: Baseline mean platelet volume is a strong predictor of major and life-threatening bleedings after transcatheter aortic valve replacement
Source: PLoS One. 2021 Nov 30;16(11):e0260439. doi: 10.1371/journal.pone.0260439 (PMC8631672; doi:10.1371/journal.pone.0260439)
Supplement: S1 Table — (DOCX) [file pone.0260439.s001.docx]

**Table S1. Procedural characteristics.**

| **Variables** | **MPV before TAVR** | | ***p* value** |
| --- | --- | --- | --- |
|  | **≤10 fL**  **L-MPV**  **(n = 398)** | **>10 fL**  **H-MPV**  **(n = 713)** |  |
| Transfemoral approach | 367 (92.2) | 650 (91.2) | 0.55 |
| Transcarotid approach | 17 (4.3) | 36 (5.0) | 0.56 |
| Sheath size |  |  |  |
| 14 F | 219 (55.0) | 407 (57.1) | 0.44 |
| 16 F | 68 (17.1) | 140 (19.6) | 0.27 |
| 18 F | 91 (22.9) | 131 (18.4) | 0.14 |
| 20 F or more | 18 (4.5) | 27 (3.8) | 0.82 |
| Balloon aortic valvuloplasty | 34 (8.5) | 43 (6.0) | 0.11 |
| Sapien | 240 (60.3) | 429 (60.2) | 0.95 |
| CoreValve | 155 (38.9) | 285 (40.0) | 0.75 |
| Acurate | 2 (0.5) | 1 (0.1) | 0.29 |
| Post-dilatation | 47 (11.8) | 87 (12.2) | 0.47 |

Data are expressed as n (%).

*Abbreviations*: H-MPV = high mean platelet volume; L-MPV = low mean platelet volume; MPV = mean platelet volume; TAVR = transcatheter aortic valve replacement
