# Supplementary material for: Baseline mean platelet volume is a strong predictor of major and life-threatening bleedings after transcatheter aortic valve replacement
Source: PLoS One. 2021 Nov 30;16(11):e0260439. doi: 10.1371/journal.pone.0260439 (PMC8631672; doi:10.1371/journal.pone.0260439)
Supplement: S2 Table — (DOCX) [file pone.0260439.s002.docx]

**Table S2. Patients with and without MPV ≤10 fL in the matched cohort for dual antiplatelet and anticoagulant therapies.**

| **Variables** | **Baseline MPV** | | ***p* value** |
| --- | --- | --- | --- |
|  | **≤10 fL**  **L-MPV**  **(n = 389)** | **>10 fL**  **H-MPV**  **(n = 389)** |  |
| **Demographic characteristics** | | | |
| Age – years | 82.7 ± 7.2 | 82.5 ± 7.4 | 0.67 |
| Male sex – n (%) | 175 (45) | 185 (48) | 0.52 |
| BMI – kg/m^2^ | 26.5 ± 5.3 | 27.1 ± 6.1 | 0.21 |
| Logistic EuroSCORE – % | 18.2 ± 12.6 | 19.6 ± 14.3 | 0.18 |
| EuroSCORE 2 – % | 5.3 ± 6.0 | 6.0 ± 6.5 | 0.12 |
| STS mortality – % | 6.3 ± 6.4 | 6.6 ± 5.4 | 0.55 |
| **Cardiovascular risk factors** | | | |
| Current smoking – n (%) | 14 (3.6) | 20 (5.1) | 0.38 |
| Hypertension – n (%) | 317 (81.5) | 322 (82.8) | 0.71 |
| Dyslipidemia – n (%) | 226 (58.1) | 231 (59.4) | 0.77 |
| Diabetes mellitus – n (%) | 127 (32.6) | 121 (31.1) | 0.70 |
| **Medical history** | | | |
| Coronary artery disease – n (%) | 198 (50.9) | 181 (46.5) | 0.25 |
| Peripheral artery disease – n (%) | 105 (27.0) | 104 (26.7) | 1.00 |
| Atrial fibrillation – n (%) | 172 (44.2) | 158 (40.6) | 0.35 |
| Heart failure – n (%) | 164 (42.3) | 166 (42.7) | 0.97 |
| Chronic kidney disease – n (%)* | 61 (15.7) | 78 (20.1) | 0.13 |
| Chronic obstructive pulmonary disease – n (%) | 48 (12.3) | 63 (16.2) | 0.15 |
| History of cancer – n (%) | 95 (24.4) | 98 (25.2) | 0.87 |
| **Echocardiographic parameters before TAVR** | | | |
| LVEF, % | 53.2 ± 14.6 | 52.0 ± 15.3 | 0.25 |
| LV mass, g/m^2^ | 128 ± 35 | 140 ± 42 | **<0.001** |
| LVEDD, mm | 49.1 ± 8.5 | 50.5 ± 8.5 | **0.02** |
| LVESD, mm | 33.4 ± 9.1 | 35.3 ± 10.0 | **0.01** |
| Mean aortic pressure gradient, mmHg | 46.6 ± 14.5 | 47.4 ± 14.1 | 0.43 |
| AVA, cm^2^ | 0.76 ± 0.24 | 0.74 ± 0.22 | 0.27 |
| Systolic PAP, mmHg | 40.6 ± 14.9 | 40.8 ± 13.6 | 0.87 |
| **Prehospital antithrombotic management** | | | |
| Dual APT – n (%) | 99 (25.5) | 95 (24.4) | 0.78 |
| Anticoagulant therapy – n (%) | 149 (38.3) | 138 (35.5) | 0.46 |
| **Discharge antithrombotic medication** | | | |
| Aspirin – n (%) | 359 (92.3) | 364 (93.6) | 0.58 |
| Clopidogrel – n (%) | 230 (59.1) | 231 (59.4) | 1.00 |
| Dual APT – n (%) | 223 (57.3) | 223 (56.3) | 1.00 |
| Anticoagulant therapy – n (%) | 165 (42.4) | 165 (42.4) | 1.00 |

Otherwise specified, data are presented as mean ± SD.

* Chronic kidney disease was defined by a creatinine level >150 µmol/L

*Abbreviations*: APT = antiplatelet therapy; AVAi = aortic valve area indexed; BMI = body mass index; H-MPV = high mean platelet volume; L-MPV = low mean platelet volume; LV = left ventricle; LVEDD = left ventricular end-diastolic diameter; LVEF = left ventricle ejection fraction; LVESD = left ventricular end-systolic diameter; MPV = mean platelet volume; PAP = pulmonary arterial pressure; STS = society of thoracic surgeons; TAVR = transcatheter aortic valve replacement
