# Supplementary material for: Baseline mean platelet volume is a strong predictor of major and life-threatening bleedings after transcatheter aortic valve replacement
Source: PLoS One. 2021 Nov 30;16(11):e0260439. doi: 10.1371/journal.pone.0260439 (PMC8631672; doi:10.1371/journal.pone.0260439)
Supplement: S3 Table — (DOCX) [file pone.0260439.s003.docx]

**Table S3. Sensitivity analyses assessing association of major and life-threatening bleeding complications during the first year after TAVR with MPV ≤10 fL.**

|  | **MLBCs**  **sHR (95% CI)** | **p value** |
| --- | --- | --- |
| MPV ≤10 fL |  |  |
| Unadjusted | 1.33 (1.03 - 1.74) | **0.03** |
| Adjusted* | 1.34 (0.98 - 1.84) | 0.07 |

* Adjusted for DAPT and anticoagulants therapy at discharge.

*Abbreviations*: MLBCs = major and life-threatening bleeding complications, MPV = mean platelet volume.
