# Supplementary material for: Baseline mean platelet volume is a strong predictor of major and life-threatening bleedings after transcatheter aortic valve replacement
Source: PLoS One. 2021 Nov 30;16(11):e0260439. doi: 10.1371/journal.pone.0260439 (PMC8631672; doi:10.1371/journal.pone.0260439)
Supplement: S4 Table — (DOCX) [file pone.0260439.s004.docx]

**Table S4. Outcomes during first year after TAVR in the subgroup of patients treated with new generation devices (Sapien 3, Evolut-Pro and Accurate-Neo).**

| **Variables** | **MPV before TAVR** | | ***p* value** |
| --- | --- | --- | --- |
|  | **≤10 fL**  **L-MPV**  **(n = 217)** | **>10 fL**  **H-MPV**  **(n = 401)** |  |
| **Bleeding events** | | | |
| Major and life-threatening bleeding <1 year | 48 (22.1) | 69 (17.2) | 0.27 |
| Major and life-threatening bleeding <30 days | 43 (19.8) | 61 (15.2) | 0.26 |
| Major bleeding <30 days | 34 (15.7) | 36 (9.0) | **0.022** |
| Major bleeding between 30 days and 1 year | 14 (6.5) | 16 (4.0) | 0.42 |
| Life threatening bleeding <30 days | 14 (6.5) | 28 (7.0) | 0.94 |
| Life-threatening between 30 days and 1 year | 4 (1.8) | 6 (1.5) | 0.90 |
| Bleeding requiring RBC transfusion >2 U | 48 (22.1) | 63 (15.7) | 0.08 |
| Minor bleeding | 41 (18.9) | 74 (18.5) | 0.96 |
| Access site vascular complications | 84 (38.7) | 116 (29.0) | **0.018** |
| **Mortality** | | | |
| Death from any cause | 30 (13.8) | 46 (11.5) | 0.64 |
| Cardiovascular death | 18 (8.3) | 15 (6.2) | 0.44 |
| **Ischemic events** | | | |
| Myocardial infarction | 8 (3.7) | 11 (2.7) | 0.52 |
| Stroke | 16 (7.4) | 28 (7.0) | 0.86 |

Data are expressed as n (%).

*Abbreviations*: H-MPV = high mean platelet volume; L-MPV = low mean platelet volume; MPV = mean platelet volume; RBC = red blood cells; TAVR = transcatheter aortic valve replacement
